# Dietary Fibre Modulates Body Composition, Blood Glucose, Inflammation, Microbiome, and Metabolome in a Murine Model of Periodontitis

**DOI:** 10.3390/nu17071146

**Published:** 2025-03-26

**Authors:** Thilini Jayasinghe, Josie Jenkins, Nidhi Medara, Phannaphat Choowong, Gangani Dharmarathne, Fay Kong, Hanna Cho, Se Hun Kim, Yuchen Zhang, Ricardo Franco-Duarte, Joerg Eberhard, Axel Spahr

**Affiliations:** 1 The Charles Perkins Centre, Faculty of Medicine and Health, University of Sydney, Camperdown, NSW 2050, Australia; josie.jenkins@sydney.edu.au (J.J.); phannaphat.choowong@sydney.edu.au (P.C.); joerg.eberhard@sydney.edu.au (J.E.); 2 School of Dentistry, Faculty of Medicine and Health, University of Sydney, Surry Hills, NSW 2006, Australia; nidhi.medara@sydney.edu.au (N.M.); fkon0350@uni.sydney.edu.au (F.K.); hcho2932@uni.sydney.edu.au (H.C.); skim3557@uni.sydney.edu.au (S.H.K.); yzha0712@uni.sydney.edu.au (Y.Z.); axel.spahr@sydney.edu.au (A.S.); 3 Australian Laboratory Services Global, Water and Hydrographic, Hume, ACT 2620, Australia; ganganidharmarathne@gmail.com; 4 Centre of Molecular and Environmental Biology, Department of Biology, University of Minho, 4710-057 Braga, Portugal; ricardofilipeduarte@bio.uminho.pt

**Keywords:** dietary fibre, periodontitis, microbiome, metabolomics, inflammation

## Abstract

**Background:** Dietary fibre plays a crucial role in metabolic regulation, inflammation, and microbiome composition. However, its impact on systemic and oral health, particularly in periodontitis, remains unclear. This study investigated the effects of high- and low-fibre diets on body composition, glycaemic control, inflammation, microbiome, and metabolome in a murine model of experimental periodontitis. **Methods:** Thirty-six male C57BL/6 mice were randomised to a high-fibre (40% fibre) or low-fibre (5% fibre) diet for eight weeks. Body weight, fat mass, lean mass, fasting blood glucose, serum inflammatory markers, alveolar bone loss, and root length were assessed. Oral and faecal microbiome composition was analysed using 16S rRNA sequencing. Metabolomic and short-chain fatty acid (SCFA) profiling was conducted using liquid chromatography–mass spectrometry (LC-MS). **Results:** Mice on the high-fibre diet exhibited significantly lower body weight (*p* < 0.0001), fat mass (*p* = 0.0007), and lean mass (*p* < 0.0001) compared to the low-fibre group. Fasting blood glucose levels were significantly lower in the high-fibre group (*p* = 0.0013). TNF-α and IFN-γ levels were significantly elevated in the low-fibre group (*p* < 0.0001), suggesting a heightened pro-inflammatory state. While alveolar bone loss and root length did not differ significantly, microbiome analysis revealed distinct bacterial compositions (PERMANOVA, *p* < 0.05), with fibre-fermenting taxa enriched in high-fibre-fed mice. Metabolomic analysis identified 19 significantly altered metabolites, indicating dietary adaptations. **Conclusions:** A high-fibre diet improves glycaemic control, reduces systemic inflammation, and alters microbial and metabolic profiles in experimental periodontitis. These findings highlight dietary fibre’s role in modulating metabolic and inflammatory pathways relevant to periodontal and systemic diseases.

## 1. Introduction

Periodontal diseases, particularly periodontitis, represent a significant global health challenge, affecting millions worldwide. Periodontitis is characterised by the pathological loss of tooth-supporting structures, including the periodontal ligament and alveolar bone, and is a major contributor to oral health disparities [1]. Beyond its local effects, periodontitis is increasingly recognised for its systemic implications, with associations among metabolic disorders, cardiovascular diseases, and immune dysregulation [2]. Between 2011 and 2020, the prevalence of periodontitis was estimated to be around 62%, with severe periodontitis affecting 23.6% of the adult population [3]. Given its widespread impact and potential consequences on systemic health, effective prevention and management strategies are urgently needed.

A key factor in the pathogenesis of periodontitis is the dysbiotic oral microbiome, which fosters a chronic inflammatory environment [4]. Periodontal pathogens such as *Porphyromonas gingivalis, Treponema denticola*, and *Tannerella forsythia* form biofilms, producing virulence factors that trigger an immune response [5]. This leads to the release of pro-inflammatory cytokines, including Tumor Necrosis Factor-alpha (TNF-α), Interleukin-1 (IL-1), and matrix metalloproteinases (MMPs), exacerbating tissue destruction [6,7,8,9,10]. Inflammation-driven bone loss follows, with alveolar bone resorption contributing to tooth loss and disease progression [11].

Interestingly, dietary factors have emerged as potential modulators of periodontitis severity, particularly through their influence on inflammation and the gut microbiome [12,13]. Dietary fibre is widely recognised for its role in modulating systemic metabolism, gut microbiota composition, and inflammatory responses [14,15]. It is fermented by gut microbiota, producing short-chain fatty acids (SCFAs) such as acetate, butyrate, and propionate [16], which have been shown to exert anti-inflammatory and immunomodulatory effects [17,18]. Given the well-established bidirectional relationship between systemic inflammation and oral health [19], it is plausible that dietary fibre may influence periodontal health through its systemic effects.

SCFAs, particularly butyrate, modulate immune responses through multiple mechanisms. They inhibit histone deacetylase (HDAC), leading to histone acetylation and the transcription of anti-inflammatory genes [20,21]. SCFAs also activate G-protein-coupled receptors (GPCRs) on immune cells, including GPR41 and GPR43 [22,23]. This interaction fosters the production of anti-inflammatory cytokines, such as IL-10, while supressing pro-inflammatory cytokines like TNF-alpha and IL-6 and mediators like Nitric Oxide Synthase (NOS) [24]. Furthermore, SCFAs enhance gut barrier function, reducing systemic endotoxemia and inflammation, while also playing a role in glucose homeostasis and metabolic regulation [25,26]. These systemic effects suggest that dietary fibre intake may influence not only body composition and glycaemic control but also the inflammatory processes underlying periodontitis progression.

Although previous studies have indicated that certain dietary fibres, such as β-glucans, may have protective effects against periodontitis [12], the mechanistic relationship between dietary fibre, inflammation, the microbiome, and periodontal disease remains poorly understood [13]. Understanding this interplay is crucial for developing diet-based interventions for periodontal disease prevention and management.

This study aimed to investigate the impact of a high-fibre diet on periodontal health, inflammation, body composition, microbiome, and metabolome in a murine model of periodontitis. Specifically, we sought to assess the effect of a high-fibre diet on periodontal bone loss, evaluate serum inflammatory markers in mice fed high- and low-fibre diets, characterise gut and oral microbiota changes in response to dietary fibre intake, and analyse SCFAs and microbial-derived metabolites to explore potential mechanistic pathways linking dietary fibre and inflammation. We hypothesised that a high-fibre diet would mitigate periodontitis severity by exerting systemic anti-inflammatory effects, leading to reduced alveolar bone loss and a distinct gut and oral microbiome composition compared to a low-fibre diet.

## 2. Materials and Methods

### 2.1. Animals and Husbandry

**Figure 1:**
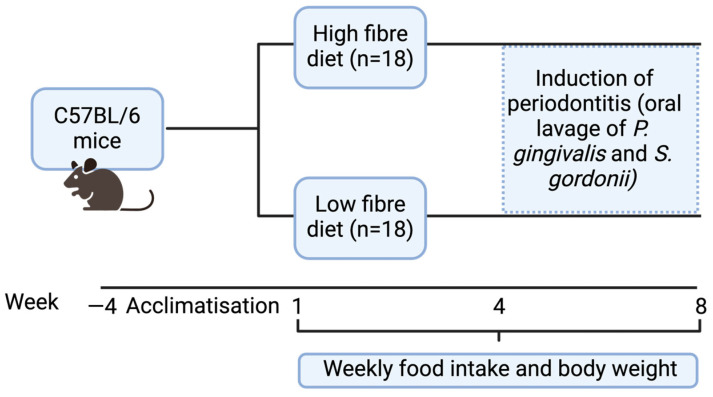
Study design for diet and periodontitis induction in C57BL/6 mice.

Thirty-six male C57BL/6 mice, aged 4 weeks upon receipt from Australian BioResources PTY Limited (ABR) (Moss Vale, Sydney, NSW, Australia), were used in this study (Figure 1). An initial acclimatisation period of 4 weeks was implemented to monitor mouse behaviour.

The sample size for each group was based on a power analysis by simulation. This analysis suggested that 18 mice per diet would provide 90% power at an alpha level of 0.05. The effect size considered was a 20% reduction in bone loss in the high-fibre group compared to the low-fibre group relative to the intercept. This estimation was based on previous research conducted by Parvaneh et al. [27], which reported a mean bone loss of 1.17 and a standard deviation of 0.21 in infected mice on a chow diet with a macronutrient composition of 19% protein, 64% carbohydrate, and 17% fat, at an energy density of 14 kJ/g.

Mice were randomly assigned to either the high-fibre or low-fibre diet group using a computer-generated randomisation table (Random Number Generator). No baseline characteristics, such as weight or age, were considered during the randomisation process.

Three mice per cage were housed in the Charles Perkins Centre at the University of Sydney, in a room with controlled temperature (22  ±  2 °C) and humidity (40–45%). The light/dark cycle was set to 12 h light and 12 h dark throughout all experimental periods. Diets (experimental diet intervention) and water were provided ad libitum, including during the acclimatisation phase (standard chow rodent diet). Prior to euthanasia, all mice underwent a 6 h fasting period with ad libitum access to water. The mice were treated according to the ARRIVE guidelines [28], and all procedures were conducted in accordance with institutional guidelines for animal research. Approval for this study was obtained from the Animal Ethics Committee and Animal Research Authority of the University of Sydney (protocol number 2021/1940), ensuring compliance with international laws and policies.

### 2.2. Diets

Diets were sourced from Speciality Feeds (Glen Forrest, WA, Australia) and were stored in a refrigerator at 4 °C to protect them from light and moisture. Two different diets containing resistant starch were prepared by incorporating Gem Star resistant starch (RS) and Guar Gum in varying ratios: a high-fibre diet consisting of 20% RS and 20% Guar Gum and a low-fibre diet containing 1.15% RS and 1.15% Guar Gum. The digestible energy contents of the high-fibre and low-fibre diets were 10.7 MJ/kg and 16.8 MJ/kg, respectively. These experimental diets were provided from week 1 to week 8 (Figure 1). Detailed compositions of the two diets are provided in the Supplementary Table S1. To ensure consistency in intake, diets were weighed twice a week. In cages where intake was lower, food was cut into 1 cm rounds to facilitate holding and consumption by the mice.

### 2.3. Food Intake

Once a week throughout the 8-week experimental period, a 24 h diet intake was measured. The mice diets were weighed and placed in a food hopper, then reweighed after a 24 h period. The difference between the weights before and after 24 h was calculated, and this value was divided by the number of mice in each cage (3 mice per cage) to estimate the food intake per mouse.

### 2.4. Induction of Experimental Periodontitis

#### Preparation of Bacterial Culture and Oral Infection

*Porphyromonas gingivalis* strain ATCC 3327 was cultured on a Brain Heart Infusion (BHI) (Thermo Fisher Scientific, Scoresby, VIC, Australia) agar plate supplemented with 0.5% *w*/*v* of yeast extract, 0.05% *w*/*v* of L-Cysteine, 4% *v*/*v* of sheep blood, 0.2% *w*/*v* of menadione (0.5 mg/mL), and 1% *w*/*v* of hemin (0.5 mg/mL). Agar plates with bacteria grown were incubated anaerobically at 37 °C for 96 h in a Bugbox anaerobic chamber (Baker, Sanford, MA, USA) with a gas composition of 10% CO_2_, 5% H_2_, and 85% N_2_. A single colony was isolated from the plate and was grown in BHI broth (excluding sheep blood) at 37 °C for 24 h.

*Streptococcus gordonii* strain ATCC 10558 was cultured on a Brain Heart Infusion (BHI) agar plate and incubated at 37 °C for 24 h in 10% CO_2_. A single colony was isolated from the agar plate and inoculated in BHI broth supplemented with 0.3% (*w*/*v*) of yeast extract, and then incubated for 24 h at 37 °C.

The concentration of *P. gingivalis* in the broth media was determined at the mid-log phase at a wavelength of 500 nm (OD  =  0.5), while the concentration of *S. gordonii* was determined at a wavelength of 600 nm (OD  =  0.6). The bacteria cultures were harvested by centrifugation and resuspension in a 1:1 (*v*/*v*) ratio in 2% (*w*/*v*) of Carboxyl Methyl Cellulose (CMC) (Alpha Chemicals, Wetherill Park, NSW, Australia) in Phosphate-Buffered Solution (PBS) (Sigma-Aldrich, St. Louis, MO, USA).

All mice, aged 12 weeks (week 4 of the experiment, Figure 1), were infected via oral lavage with 0.5 mL of a suspension containing *P. gingivalis* and *S. gordonii* (0.2 mL each, at a concentration of 10^10^–10^11^ CFU/mL) daily for four weeks. The oral lavage was administered using a 1 mL laboratory pipette (Eppendorf, Hamburg, Germany).

### 2.5. Changes in Body Weight

Body weight measurements were conducted using an automatic electronic balance (Weight Pty LTD, Moorabbin, VIC, Australia) twice a week throughout the 8-week duration of the experiment. In accordance with the stipulations set forth by the Animal Ethics Committee, any mouse that experienced weight loss exceeding 15% of its initial body weight was subject to humane euthanasia through CO_2_ asphyxiation.

### 2.6. Body Composition Analysis Using EchoMRI

EchoMRI (EchoMRI, LLC, Houston, TX, USA) scans were conducted at the Pre-Imaging Core Research Facility at the University of Sydney. Each mouse underwent examination at week 4 (the final week of acclimatisation before the stated diet intervention) and week 12 (the fourth week of oral lavage before euthanasia) to determine total body mass, lean mass, and free fluid. Mice were placed in appropriately sized animal tube holders based on their body weight and were restrained from making large movements. The tube insert was secured with a Velcro strap, and the entire animal tube was inserted into the bore entrance of the EchoMRI 900 machine for scanning. The total procedure time for each mouse was approximately 2 min, after which mice were returned to their holding cage.

### 2.7. Collection of Oral and Stool Samples

Oral and stool samples were collected at 16 weeks prior to euthanasia for subsequent microbiome analysis. Oral samples were collected under Isoflurane sedation with a dose of 2% *v*/*v*. FLOQ swabs dipped and soaked in saline solution were swirled around the buccal cavity of each mouse for 60 s, resulting in the use of two swabs for 30 s each. These swabs were then placed in individual Eppendorf tubes and stored at −80 °C until further testing.

Stool samples were collected simultaneously with the oral swabs. Each mouse was isolated from its cage to produce an average 0.05 g of stool, which was collected and stored in Eppendorf tubes in −80 °C for subsequent analysis.

### 2.8. Fasting Blood Glucose Measurements

Fasting blood glucose measurements were taken at the endpoint. Diets were removed from the mouse cages 6 h prior to the blood glucose measurement, resulting in a fasting period of 6 h (8 a.m. to 2 p.m.). Mice were then removed from the holding room and transferred to a PLAS-LAB restraining device with their tails extended. A needle was carefully inserted into the lateral tail vein, approximately 2–3 cm from the tail tip. A drop of blood was applied on a glucometer (Accu-check Performa II, Accu-check, Corydon, IN, USA) to measure blood glucose levels. Pressure was applied to encourage clotting after the measurement was taken.

### 2.9. Euthanasia and Tissue Processing

At the end of the diet intervention (8 weeks), and following four weeks of oral wash procedures, mice were anesthetised using 4% *v*/*v* of Isoflurane (Isothesia NXT, Covetrus, Portland, ME, USA) administered for 3 min. Subsequently, cardiac puncture was performed, and the blood was centrifuged to isolate serum. Caecal content was collected, snap-frozen using liquid nitrogen, placed on dry ice, and stored at −80 °C until analysis.

### 2.10. Measurements of Alveolar Bone Loss

#### 2.10.1. Micro-CT Tomography

Micro-CT (U-CT uhr/MSA, MiLabs, Houten, The Netherlands) with MILabs Recon (v. 9, MiLabs, Houten, The Netherlands) was performed on the frozen detached heads at the Preclinical Imaging Facility, Charles Perkins Centre, NSW, Australia. The samples were placed in a mouse bed with the incisors facing superiorly, fixed in the position with tape and scanned with the following parameters on the micro-CT: ultra-focus magnification and accurate scan mode. This equates to the X-ray generator operated at an accelerated potential of 50 kV with a beam current of 240 μA, an exposure time of 75 ms with a primary AI 100 μm filter, 360° scan rotation, a step angle of 0.25°, and a dose estimate of 1957 mGy. The images were reconstructed at a 6–9 μm voxel size with the Imalytics Preclinical software (v. 2.1.8.5, Gremse-IT, Aache, Germany) at 6–9 microns.

#### 2.10.2. Measurement of Alveolar Bone Loss

**Figure 2:**
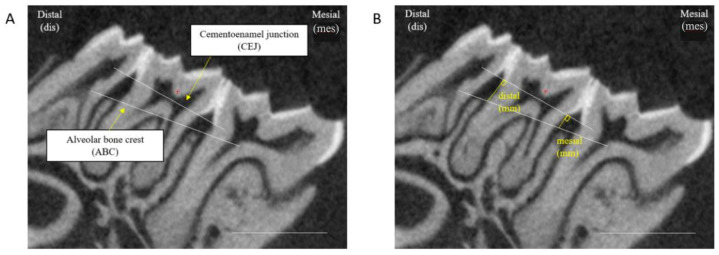
The distance between the CEJ and ABC as a measurement of alveolar bone loss (mm). (**A**): Images were reoriented such that the CEJ and ABC at the middle of the M2 molar appear at the same slice. (**B**): The distance between the CEF and ABC was measured at two sites of the M2 molar tooth.

Image analysis and measurements were performed using ImageJ (v. 1.37q). To ensure consistency, all images were reoriented such that the cementoenamel junction (CEJ) and the alveolar bone crest (ABC) at the middle of the second molar (M2) were aligned (Figure 2A). The alveolar bone loss (mm) of each jaw was determined by measuring the mesial and distal distances between the CEJ-ABC of the M2 molar, where a greater distance indicates higher alveolar bone loss and potentially poorer oral health (Figure 2B). Root length (mm) was determined by measuring the mesial and distal distances between the CEJ to the apex of the M2 molar. Measurements were taken at both the upper and lower jaws (left and right sides) for each mouse, and the average of these measurements was calculated to obtain the final alveolar bone loss value.

### 2.11. Measurement of Fasting Serum Cytokine

Serum cytokines (IFN-γ, IL-1β, IL-6, IL-10, IL-17A, and TNF-α) were measured in fasting serum samples using the Bio-Plex Pro^TM^ Mouse CytokineTh17 Panel A 6-Plex Assay kit (Bio-Rad, Hercules, CA, USA) according to the manufacturer’s instructions. All measurements were performed using a Luminex MAGPIX bead assay reader instrument (Luminex, Austin, TX, USA) and Luminex xPonent software programme (v. 4.2, Luminex, Austin, TX, USA).

### 2.12. DNA Extraction from Oral Swabs

DNA extraction from oral swabs was performed using the ZymoBiomics DNA kit (Zymo Research, Freiburg, Germany) according to the manufacturer’s instructions. Due to cost constraints, oral microbiome analysis was conducted on a subset of 23 samples (high fibre: 11, low fibre: 12), which were randomly selected for processing. Briefly, oral swab samples were collected and placed directly into ZR BashingBeads lysis tubes. These tubes were then placed in a tissue lyser and subjected to agitation for 10 min at max speed, with 2 min intervals, followed by breaks in an ice bath.

After completion of the lysis process, the contents were centrifuged, and the supernatant was collected into a filter attachment connected to a collection tube. Binding buffer was added to the collected supernatant, followed by DNA wash buffer. The DNA was then vortexed with ZymoBIOMICS DNase/RNase-Free Water and filtered through a Zymo-Spin II-μHRC Filter into a new collection tube. ZymoBIOMICS HRC Prep Solution was added to facilitate the elution of the DNA. The final DNA extract was measured for purity and concentration using a nanodrop reader, with A260/A280 and A260/A230 ratios recorded for further optimisation purposes.

### 2.13. DNA Extraction from Faeces

DNA extraction from faeces (collected before euthanasia) was performed using the Qiagen Power Soil Pro kit (Qiagen, Hilden, Germany), according to the manufacturer’s instructions. Faecal microbiome analysis was conducted on 31 samples (high fibre: 14, low fibre: 17), all of which were included in the analysis. Briefly, faecal samples stored at −80 °C were thawed and placed into Qiagen PowerBead Pro Tubes, followed by vortexing with the first buffer solution. After incubation at 60 °C for 10 min, the PowerBead Pro Tubes were subjected to tissue lysis using a tissue lyser for 10 min. Following the completion of tissue lysis, the samples were centrifuged, and the supernatant was extracted using a pipette. DNA was purified according to the Qiagen protocol and incubated on the MB Spin Column filter membrane for an additional minute before the elution of DNA. The DNA concentration (ng/μL) was determined using a nanodrop, as well as a measurement of the A260/280 and A260/230 ratios. DNA was diluted to achieve a concentration of 5–10 ng in 20 μL before being sent to the Ramaciotti Institute (University of New South Wales, Australia) for 16SrRNA gene amplicon sequencing.

### 2.14. Microbiota Analysis Using 16S rRNA Gene Amplicon Sequencing Technique

The Ramaciotti Institute performed the 16S rRNA gene amplicon sequencing for both oral and faecal DNA samples. The microbial community was profiled using the 16S rRNA V4 region (nucleotide position 515–806) with Illumina MiSeq (Illumina, Inc., San Diego, CA USA), producing 250 base-pair paired-end reads. Sequencing data were analysed using Qiime2 with standard parameters (version 2024.5) [29]. Quality control was performed using DADA2 parameters, followed by taxonomic assignment using the GreenGenes v.13.5 database for faecal samples and the Human Oral Microbiome Database (HOMD) for oral samples. Representative sequences were aligned using MAFFT (v. 7.511) and used to build a phylogenetic tree with fasttree.

The sequence analysis output of 16S rRNA amplicon data from QIIME2 was imported into R (version 4.4.0) for downstream analysis. Relative abundance, alpha, and beta diversity were derived using the PhyloSeq package (version 1.48.0) [30].

### 2.15. Targeted LCMS Analysis in Mice Faecal Samples Using LC-Orbitrap-MS

Polar metabolite analysis using liquid chromatography–mass spectrometry (LC-MS) was conducted at NCRIS-enabled Metabolomics Australia, University of Melbourne, with funding support from BioPlatforms Australia. Due to cost constraints, faecal polar metabolite analysis was conducted on a subset of 12 samples (high fibre: 6, low fibre: 6). Sample analysis was performed using LC-Orbitrap-MS as a blind study, aiming to identify 113 polar metabolites.

Upon arrival of the sample in Metabolomic Australia, the protocol proceeded as follows: 30 mg of each sample was placed into an Eppendorf tube, along with 500 µL of ice-cold extraction/lysis solution (4:1 Methanol: water containing 3 µM ^13^C of sorbitol, 3 µM ^13^C of ^15^N-valine, and 3 µM ^13^C of leucine). The faecal pellet was homogenised at 1200 RPM in a Thermomix while being maintained at 4 °C. After 20 min, the pellets were broken through the pipette tip, and the homogenisation process was repeated for an additional 10 min. Once the faecal pellets were evenly homogenised and broken down, the samples were centrifuged at 14,100× *g* at 4 °C for 10 min. The supernatant was then transferred into a new 1.5 mL tube, and 5 µL was injected for LCMS analysis, following the protocol described in Wang et al. study [31].

Briefly, data acquisition was performed using Thermo Tracefinder (V4.1) (General Quan Browser). Metabolite identification was conducted using El-Maven v.0.12.1, where metabolite peaks were matched against an in-house standard library containing 550 polar metabolites. Quantification was based on the area under the curve (AUC) of detected metabolite peaks. For statistical analysis and normalisation, raw data were processed using MetaboAnalyst v5.0. Peak intensities of identified metabolites were median normalised before comparison across groups using the Statistical Analysis [One Factor] module in MetaboAnalyst.

### 2.16. Short-Chain Fatty Acids (SCFAs) Analysis

Similarly to the polar metabolite analysis, an SCFA analysis was conducted at Metabolomics Australia (University of Melbourne, Australia). SCFA analysis included 24 samples (high fibre: 12, low fibre: 12). Briefly, after the pre-treatment of High-Performance Liquid Chromatograph (HPLC) vials and glassware, approximately 30 mg of the caecal sample was weighed into a 1.5 mL Eppendorf vial. The caecum samples were mixed with 400 µL of 50% acetonitrile and 4 µL of 4-methylvaleric acid (from a 1 mM stock) to achieve a final concentration of 10 µM per sample. The mixture was vortexed for 30 s and incubated at 10 °C for 30 min on a thermomixer at 950 rpm. During the incubation, the pellets were intermittently broken using cut tips.

After incubation, the mixture was centrifuged at 14,000 rpm for 5 min at 4 °C to collect the supernatant in a fresh 1.5 mL Eppendorf vial. To facilitate analysis, 20 µL of purified ^13^C_6_-NPH-labelled SCFA mix (internal standard) was added to 40 µL of the extracted supernatant. Then, 200 mM NPH (3-nitrophenylhydrazine hydrochloride) and 120 mM EDC (1-ethyl-3-(3-dimethylaminopropyl) carbodiimide) were added to the same vial. The mixture was incubated at 40 °C for 30 min at 950 rpm on a thermomixer. Subsequently, 20 µL of 200 mM quinic acid was added for quenching, followed by another incubation at 40 °C for 30 min at 950 rpm. The mixture was reconstituted with 1.9 mL of 15% acetonitrile, bringing the total volume to 2 mL. A 5 µL aliquot of the reconstituted mixture was injected into an Agilent 6490 LC-QQQMS system for the analysis of SCFAs, following the protocol described by Gubert et al. [32].

Data acquisition was performed using Agilent MassHunter Workstation Software (v. 10.2, Agilent Technologies, Santa Clara, CA, USA). Metabolite identification was conducted using El-Maven v.0.12.1, where metabolite peaks were matched against an in-house standard library of SCFAs. Quantification was based on the AUC of detected metabolite peaks. For statistical analysis and normalisation, raw data were processed using MetaboAnalyst v5.0. The peak intensities of identified SCFAs were median normalised before comparison across groups using the Statistical Analysis [One Factor] module in MetaboAnalyst.

### 2.17. Statistical Analysis

For the comparison of body weight, body composition (fat and lean mass), fasting blood glucose, alveolar bone loss, and root length at the endpoint, independent *t*-tests were used. The Brunner–Munzel test was applied to compare fasting serum cytokines at the endpoint.

For microbiota analysis, microbial species were compared using DESeq2 to identify species enriched in each diet group. Alpha diversity indices (Shannon, Simpson, and Chao1) were compared between groups using the Wilcoxon rank-sum test. Beta diversity was assessed using permutational multivariate analysis of variance (PERMANOVA) based on the Jaccard index and Bray–Curtis dissimilarities. SCFAs were analysed using the Wilcoxon test, and False Discovery Rate (FDR) correction was conducted by the Benjamini–Hochberg test. Metabolomic data from LC-MS were analysed using the Mann–Whitney U test (Wilcoxon rank-sum test), and False Discovery Rate (FDR) correction was applied using the Benjamini–Hochberg method to identify significant differences between the high-fibre and low-fibre diet groups. Principal Component Analysis (PCA) was performed to explore variations in faecal metabolites between dietary groups. Prior to analysis, metabolite concentrations were log10-transformed and standardised using z-score normalisation to ensure comparability across different metabolite scales. PCA was conducted using scikit-learn in Python (v. 3.13), retaining two principal components (PC1 and PC2) for visualisation. All statistical analyses were conducted using R (version 4.4.0). Results were considered statistically significant at *p* < 0.05.

## 3. Results

During the study, five mice were culled due to specific circumstances. Four of the mice experienced a weight loss exceeding 15% of their initial body weight, with three cases occurring at week 5 and one at week 6. Additionally, one mouse developed a wound during week 5. These four mice were subsequently excluded from all data analyses conducted in this study. At the end of this study, a total of 14 mice in the high-fibre diet group and 17 mice in the low-fibre diet group remained and were included in the final analysis, unless otherwise mentioned.

The following table (Table 1) presents a comparison of various biological parameters between a high-fibre group and a low-fibre group at the study endpoint.

### 3.1. Resistant Starch Affected Body Weight, Fat and Lean Mass, and Fasting Blood Glucose Levels in Experimental Periodontitis Mice

**Figure 3:**
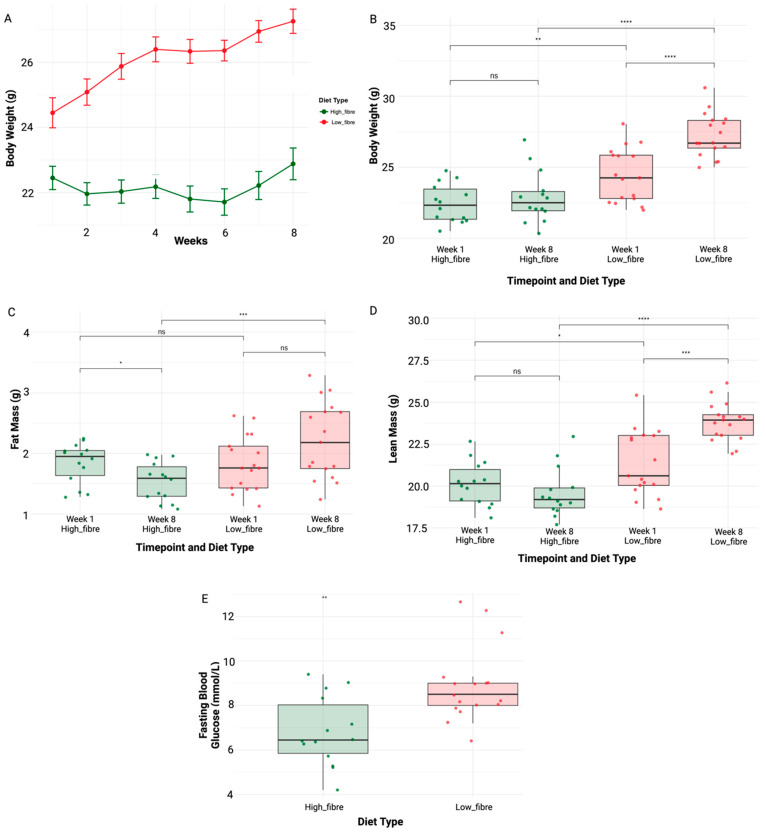
(**A**) Weekly body weight change for mice on high-fibre and low-fibre diets from baseline (week 1) to endpoint (week 8); (**B**) comparison of body weight from baseline (week 1) to endpoint (week 8) between high-fibre and low-fibre diets; (**C**) comparison of fat mass from baseline (week 1) to endpoint (week 8) between high-fibre and low-fibre diets; (**D**) comparison of lean mass from baseline (week 1) to endpoint (week 8) between high-fibre and low-fibre diets; (**E**) fasting blood glucose levels at endpoint (week 8) for mice fed low- and high-fibre diets. Statistical significance: ns = not significant; * *p* < 0.05; ** *p* < 0.01; *** *p* < 0.001; **** *p* < 0.0001.

Figure 3A depicts the weekly body weight change starting from baseline (week 1) until the endpoint (week 8). Body weights within the low-fibre diet group were along an expected and anticipated trend, compared to the high-fibre diet group who experienced limited growth, as we observed a very slight increase in the average weight of mice in the high-fibre diet group (mean and standard deviation for baseline and endpoint, respectively, 22.5 g ± 1.34 and 22.9 g ± 1.82 compared to that of the low-fibre diet group, 24.5 g ± 1.90 and 27.3 g ± 1.53). Moreover, body weight at baseline and endpoint was significantly different for the low-fibre diet (*p* = 4.2 × 10^−5^, unpaired *t*-test) but not for the high-fibre diet (Figure 3B). It is important to note that the body weight of the mice on the low-fibre diet at baseline was significantly higher than that of the high-fibre diet group (mean and standard deviation, respectively: 24.5 g ± 1.90 vs. 22.5 g ± 1.34, *p* = 0.0019, unpaired *t*-test). The high-fibre group had a significantly lower body weight (22.9 g ± 1.82) at the endpoint compared to the low-fibre group (27.3 g ± 1.53), with a high level of statistical significance (*p* = 2 × 10^−7^, unpaired *t*-test) (Table 1). Additionally, change in weight from baseline to endpoint was significantly different between the high-fibre and low-fibre diet groups (0.46 g ± 1.34 vs. 2.83 g ± 0.93, *p* = 1.12 × 10^−5^, unpaired *t*-test).

Although the baseline body weight was significantly different between the low- and high-fibre groups, the average fat mass was similar between the low-fibre and high-fibre diet groups (1.84 g ± 0.33 for high fibre and 1.84 g ± 0.45 for low fibre) (Figure 3C). However, there was a significant difference in fat mass at the endpoint between the two diet groups (high fibre vs. low fibre: average 1.53 g ± 0.32 vs. 2.22 g ± 0.63, *p* = 0.0007, unpaired *t*-test). Fat mass was significantly reduced from baseline to endpoint for the high-fibre group (1.84 g ± 0.33 vs. 1.53 g ± 0.32, *p* = 0.02, unpaired *t*-test), while it showed a non-significant increase from baseline to endpoint for the low-fibre diet (1.84 g ± 0.45 vs. 2.22 g ± 0.63, *p* = 0.055, unpaired *t*-test). Additionally, the change in fat mass from baseline to endpoint was significantly different between the high-fibre and low-fibre diet groups (0.31 g ± 0.33 vs. 0.38 g ± 0.41, *p* = 2 × 10^−5^, unpaired *t*-test).

Lean mass was significantly different between the low-fibre and high-fibre diets at the baseline (Figure 3D). Though lean mass decreased from baseline to endpoint for the high-fibre diet (20.1 g ± 1.30 vs. 19.6 g ± 1.46), it significantly increased for the low-fibre diet (21.4 ± 1.94 vs. 23.8 ± 1.15, *p* = 0.00013, unpaired *t*-test). Similarly to fat mass, there was a significant difference in lean mass at the endpoint between the two diet groups (high fibre vs. low fibre: average 19.6 g ± 1.46 vs. 23.8 g ± 1.15, *p* = 5 × 10^−9^, unpaired *t*-test). Additionally, the change in lean mass from baseline to endpoint was significantly different between the high-fibre and low-fibre diet groups (0.54 g ± 0.85 vs. 2.45 g ± 1.42, *p* = 9 × 10^−8^, unpaired *t*-test).

The endpoint fasting blood glucose data showed a notable difference between the high-fibre and low-fibre diets (Figure 3E). Specifically, the mice on the high-fibre diet exhibited significantly lower blood glucose levels compared to those on the low-fibre diet (6.83 mmol/L ± 1.56 vs. 8.92 ± 1.70, *p* = 0.0013, unpaired *t*-test).

### 3.2. Impact of High-Fibre Diet on Alveolar Bone Loss and Root Length and Inflammatory Markers

**Figure 4:**
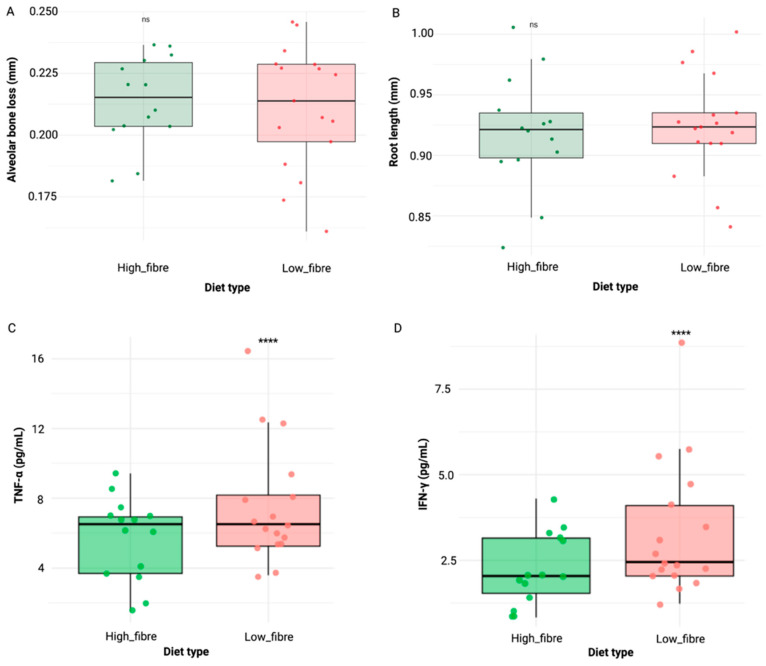
Comparison of alveolar bone loss (**A**), root length (**B**), TNF-α (**C**), and IFN-γ (**D**) at week 8 between mice fed low- and high-fibre diets. Statistical significance is indicated as follows: ns = not significant; **** *p* < 0.0001.

There was no significant difference between high-fibre and low-fibre diets in relation to alveolar bone loss and root length (Figure 4A,B). In fact, non-significant higher alveolar bone loss (0.214 ± 0.018) and lower root length (0.919 ± 0.047) were observed in the high-fibre diet group compared to the low-fibre diet group (alveolar bone loss: 0.211 ± 0.025 and root length: 0.925 ± 0.042).

With regard to the inflammatory markers, we observed significantly higher levels for TNF-α (Figure 4C) and IFN-γ (Figure 3D) in the low-fibre diet group at the 8-week mark. Specifically, TNF-α levels were significantly higher in the low-fibre (7.49 ± 3.43 pg/mL) compared to the high-fibre (5.70 ± 2.40 pg/mL) group (*p* < 0.0001, Brunner–Munzel test). Similarly, IFN-γ levels were elevated in low fibre (3.31 ± 1.96 pg/mL) vs. high fibre (2.24 ± 1.07 pg/mL) (*p* < 0.0001, Brunner–Munzel test). No significant differences were observed for IL-1β, IL-6, IL-10, and IL-17A.

### 3.3. Bacterial Profiles in the Faeces and Oral Swabs of Mice Fed High-Fibre and Low-Fibre Diets at the Endpoint

#### 3.3.1. Oral Swab Microbiome Analysis

**Figure 5:**
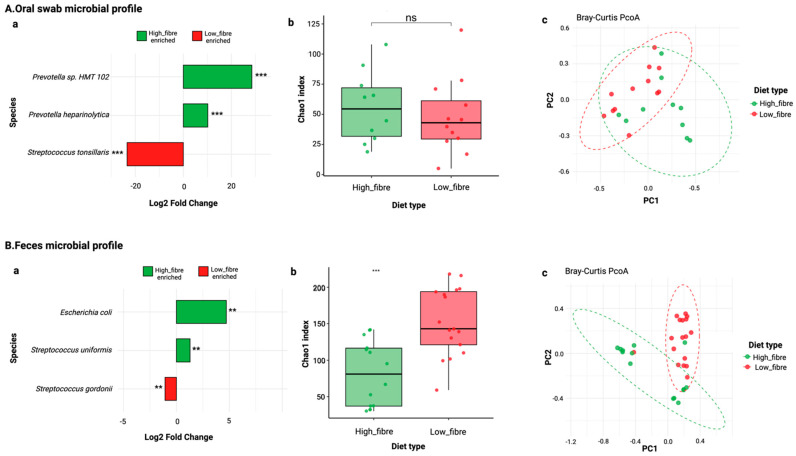
Microbial profiles identified in (**A**) oral swab samples (high fibre: *n* = 11, low fibre: *n* = 12) and (**B**) faeces samples (high fibre: *n* = 14, low fibre: *n* = 17). (**a**) Differentially abundant microbial species between high-fibre (green) and low-fibre (red) diet groups, identified using DESeq2. Log2-fold changes indicate enrichment in either diet group, with statistical significance denoted by asterisks. (**b**) Alpha diversity analysis based on the Chao1 index, comparing species richness between high-fibre and low-fibre diet groups. (**c**) Beta diversity analysis using Principal Coordinates Analysis (PCoA) based on Bray–Curtis dissimilarities, illustrating significant differences in microbial community compositions between diet groups. Each point represents an individual sample, with ellipses indicating group clustering. Statistical significance: ns = not significant; ** *p* < 0.01; *** *p* < 0.001.

Differential abundance analysis of the oral microbial profile using DESeq2 identified several taxa that were significantly enriched in each group (Figure 5A(a)). Species such as *Prevotella* sp. *HMT 102* and *Prevotella heparinolytica* were significantly enriched in the high-fibre diet group, while *Streptococcus tonsillaris* was significantly more abundant in the low-fibre diet group (Figure 5A(a)). The log2-fold change values indicated substantial shifts in microbial taxa based on dietary conditions.

None of the alpha diversity indices (Shannon, Simpson, and Chao1) showed significant differences between diet groups (Chao1: Figure 5A(b)). Beta diversity analysis using Bray–Curtis dissimilarity (Figure 5A(c)) revealed significant differences between the diet groups, as illustrated in the PCoA plot (PERMANOVA, *p* = 0.013).

#### 3.3.2. Gut Microbiome Analysis

Differential abundance analysis showed a significant enrichment of several taxa in the different diet groups. Differential abundance analysis using DESeq2 identified that *Escherichia coli* and *Streptococcus uniformis* were more abundant in the high-fibre diet group, while *Streptococcus gordonii* was significantly enriched in the low-fibre diet group (Figure 5B(a)).

Alpha diversity assessed using Shannon and Simpson indices showed no significant difference between diet groups for Shannon and Simpson indices. However, Chao1, which measures species richness, was significantly different between groups (*p* = 0.00098) (Figure 5B(b)), indicating that the low-fibre diet group harboured a higher number of observed species. Beta diversity analysis using Bray–Curtis dissimilarity revealed distinct microbial community compositions between diet groups (PERMANOVA, *p* = 0.001) (Figure 5B(c)).

### 3.4. Caecum SCFAs and Faecal Polar Metabolite Analysis at the Endpoint

**Figure 6:**
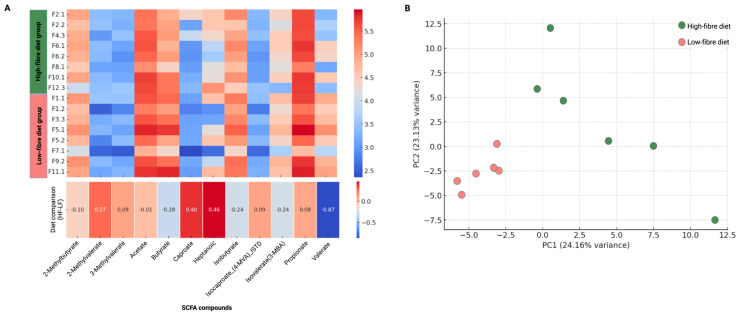
(**A**) Short-chain fatty acid (SCFA) profiles in high-fibre and low-fibre diet groups. Individual sample values were plotted in a heatmap, with samples grouped by dietary conditions. To directly compare high-fibre and low-fibre diets, the mean SCFA levels for each group were calculated, and the difference was computed and represented at the bottom (SCFA values were log10-transformed to normalise differences in magnitude across compounds). (**B**) Visualisation of variation in faecal metabolite profiles between high-fibre and low-fibre diet groups using Principal Component Analysis (PCA). All concentrations were log-transformed (log10) and standardised using z-score normalisation.

Among the SCFAs analysed (high fibre: *n* = 8, low fibre: *n* = 8), including 2-Methylbutyrate, 2-Methylvalerate, 3-Methylvalerate, acetate, butyrate, Caproate, Heptanoic acid, Isobutyrate, Isocaproate (4-MVA) ISTD, Isovalerate (3-MBA), propionate, and valerate, only valerate levels showed a significant difference between diet groups (Wilcoxon test, *p* = 0.0029). However, this difference was not statistically significant after FDR correction (adjusted *p* = 0.08) (Figure 6).

Of the 118 metabolites included in the analysis (high fibre: *n* = 6, low fibre: *n* = 6), 19 metabolites—Adenosine monophosphate, Uridine 5′-monophosphate, Deoxyinosine, Cytidine, Ornithine, propionic acid, Pyroglutamic acid, xanthine, Azelaic acid, Suberic acid, Citrulline, Threonic acid, Galactonolactone, D-Glucurono-6,3-lactone, Inosine, Gluconic acid, Phosphate, Alpha-Tocopherol, and Erythritol—were significantly different between diet groups after FDR correction (Supplementary Table S2). The PCA plot revealed a clear distinction between the low-fibre and high-fibre groups, with samples clustering separately along Principal Component 1 (PC1), which accounted for 24.16% of the total variance. Low-fibre diet samples were primarily positioned on the left side of the plot, while high-fibre samples clustered on the right, indicating that PC1 captures the main metabolic differences between dietary groups. Principal Component 2 (PC2), explaining 13.13% of the variance, contributed to additional variation within groups but did not strongly separate the dietary conditions.

## 4. Discussion

In this study, we observed significant differences in body composition, metabolic parameters, inflammation, and microbiome profiles between mice fed a high-fibre and low-fibre diet. The high-fibre group exhibited significantly lower body weight, fat mass, and lean mass at the study endpoint, with minimal weight gain over the experimental period. Notably, fasting blood glucose levels were significantly lower in the high-fibre group, while inflammatory markers, including TNF-α and IFN-γ, were significantly elevated in the low-fibre group, suggesting a more pronounced pro-inflammatory response associated with a low-fibre diet. Microbiome and metabolomic analyses revealed distinct differences in bacterial composition between diet groups. Collectively, these findings highlight the multifaceted impact of dietary fibre on body composition, glucose regulation, inflammation, and microbiome composition in experimental periodontitis mice.

Our results demonstrated that mice on the low-fibre diet exhibited significantly higher body weight, fat mass, and lean mass compared to those on the high-fibre diet. This finding aligns with previous studies showing that high-fibre diets are typically associated with lower caloric intake, reduced energy absorption, and enhanced satiety, all of which contribute to lower body weight gain [33,34,35]. Dietary fibre, particularly resistant starch, has been shown to modulate gut hormones such as peptide YY and glucagon-like peptide-1 (GLP-1), which regulate appetite and energy homeostasis [36,37]. The reduced fat mass observed in the high-fibre group could be attributed to increased microbial fermentation, leading to the production of SCFAs that influence lipid metabolism and adiposity [38,39].

Interestingly, despite significant weight differences, lean mass was also lower in the high-fibre group, which may be related to reduced overall energy intake and nutrient absorption. However, it is also possible that the high-fibre diet (40%) was excessively restrictive, leading to growth retardation or metabolic stress, which could have masked some of the protective effects hypothesised in this study, particularly the expected reduction in alveolar bone loss in the high-fibre diet group. These findings suggest that while high-fibre intake offers metabolic benefits, excessive fibre consumption may have unintended consequences. Future studies should investigate optimal fibre intake levels that maximise metabolic advantages while preventing growth impairments and further explore its impact on oral health.

Fasting blood glucose levels were significantly lower in the high-fibre diet group, further supporting the well-documented role of dietary fibre in improving glycaemic control [40,41]. Fibre-rich diets slow glucose absorption, enhance insulin sensitivity, and promote the production of SCFAs, which contribute to improved glucose metabolism [41,42,43,44]. Additionally, PYY and GLP-1, which were likely upregulated in response to high-fibre intake [37], play a crucial role in modulating inflammation, bone metabolism, and immune responses, all of which are relevant to periodontal health [45]. GLP-1 receptor agonists have been shown to enhance osteoblast proliferation, counteract bone loss, and reduce periodontal inflammation, while PYY has been implicated in immune regulation and epithelial barrier maintenance, potentially influencing oral and periodontal health [45,46].

TNF-α and IFN-γ were significantly elevated in the low-fibre diet group, indicating a more pro-inflammatory systemic state. Previous research has demonstrated that low-fibre diets can lead to increased gut permeability and endotoxemia, which in turn trigger systemic inflammation [47,48]. Conversely, high-fibre diets are known to promote anti-inflammatory effects through gut microbial fermentation and SCFA production, which modulate immune responses [43,49]. Butyrate, for example, has been shown to support the differentiation of regulatory T cells (Tregs) and suppress excessive immune activation, which could contribute to reduced systemic inflammation and periodontal protection [43].

Beta diversity analysis confirmed significant differences in microbial community compositions between diet groups, emphasising the critical role of diet in shaping gut and oral microbiota. In the oral microbiome, *Prevotella* sp. *HMT* and *Prevotella heparinolytica* were significantly enriched in the mice in the high-fibre diet group, while *Streptococcus tonsillaris* [49,50,51], a species linked to oral inflammation [52], was more abundant in the low-fibre diet group [53]. While *Prevotella* species are commonly known for their fibre-fermenting capacity, certain strains, including *Prevotella heparinolytica*, have been associated with pro-inflammatory responses, suggesting that their enrichment in the high-fibre diet group may not necessarily indicate a purely beneficial effect [54,55]. Similarly, gut microbiome analysis further demonstrated significant enrichment of *E. coli* and *Streptococcus uniformis* in the high-fibre diet group, both of which have been implicated in an inflammatory condition [56,57], whereas *Streptococcus gordonii,* an initial coloniser in periodontal disease [58], was significantly higher in the low-fibre diet group. These findings suggest that while a high-fibre diet can reshape the microbiome, its effects on inflammation are more complex than anticipated. One possible explanation is that high-fibre intake led to weight loss, which could have acted as a physiological stressor, thereby altering microbial ecology in a way that favoured specific inflammatory taxa [55]. Future studies should investigate whether fibre-induced microbiome shifts contribute to inflammation under conditions of significant weight loss.

While dietary fibre has well-established effects on gut microbiota composition and metabolite production, our study did not observe a clear protective impact of a high-fibre diet on periodontal outcomes. One potential explanation is that metabolic stress induced by weight loss in the high-fibre group may have counteracted some of the expected periodontal benefits. However, emerging evidence suggests that the gut microbiome and its metabolites play a crucial role in periodontal disease modulation, raising the possibility that dietary fibre may exert indirect effects through gut–immune interactions [59,60,61]. SCFAs, particularly butyrate and propionate, are known to modulate inflammation, enhance immune homeostasis, and support epithelial barrier integrity, all of which are crucial for periodontal health [62,63]. Additionally, beneficial bacterial genera such as Bifidobacterium and Akkermansia, both associated with anti-inflammatory effects, were significantly altered in response to dietary fibre intake [64,65]. While these microbial and metabolic changes may have systemic implications, their direct influence on periodontal disease progression remains unclear. Future studies should investigate whether dietary fibre-mediated microbiome shifts translate into protective effects against periodontal inflammation and bone loss. Furthermore, exploring the gut–oral axis through mechanistic studies could provide deeper insights into the interplay between microbial metabolites and periodontal health.

Additionally, metabolomic analysis revealed significant differences in several faecal metabolites between diet groups, with high-fibre-fed mice exhibiting higher levels of metabolites associated with improved metabolic health, such as propionic acid and xanthine. Propionic acid, a key SCFA, has been shown to enhance insulin sensitivity, regulate lipid metabolism, and exert anti-inflammatory effects through G-protein-coupled receptors (GPR41/GPR43) and the inhibition of histone deacetylases (HDAC) [66,67]. Xanthine, a purine metabolite involved in antioxidant defence and metabolic homeostasis, was elevated in the high-fibre group. Xanthine and its derivatives have been reported to modulate immune responses, reduce oxidative stress, and influence glucose metabolism, potentially mitigating inflammatory and metabolic disturbances associated with periodontitis and systemic disease [68,69]. Furthermore, the observed differences in microbial-derived metabolites align with gut microbiota shifts between diet groups, reinforcing the role of dietary fibre in shaping host metabolism via microbial fermentation and metabolite production [43]. Collectively, these findings highlight the systemic metabolic effects of dietary fibre, suggesting potential benefits for glycaemic control and inflammation regulation beyond the gut.

However, while short-chain fatty acids (SCFAs) are typically expected to increase with dietary fibre intake due to microbial fermentation, none of the caecum SCFAs, including acetate, butyrate, and propionate, were significantly elevated in the high-fibre diet. The only SCFA showing an initial statistical difference was valerate. Valerate, though less studied than other SCFAs, has been linked to gut microbial metabolism and immune regulation [66]. The absence of significant SCFA elevation in the high-fibre group could be attributed to factors such as individual variability, microbial adaptation, or the specific fibre composition used in this study. This suggests that the metabolic benefits observed in the high-fibre group may be mediated through mechanisms beyond SCFA production, potentially involving alternative microbial metabolites or systemic metabolic adaptations.

Interestingly, alveolar bone loss and root length did not show significant differences between diet groups, although there was a non-significant trend toward greater alveolar bone loss in the high-fibre group. This unexpected finding may be explained by the lower body weight and potential stress-related effects in the high-fibre diet group, which could have masked any protective effects of dietary fibre on bone health. Previous studies suggest that gut dysbiosis and inflammation can impact bone metabolism, with SCFAs playing a role in bone resorption and immune modulation [70]. Further research is required to investigate the mechanistic pathways linking dietary fibre, gut microbiota, and alveolar bone integrity.

This study presents several strengths, including a well-controlled experimental design and comprehensive assessment of multiple biological parameters, providing a holistic perspective on the impact of dietary fibre. However, it is important to acknowledge the inherent limitations of the murine model. Although mouse models allow for controlled dietary interventions and detailed mechanistic investigations, they do not fully replicate the complexity of human periodontal disease, including differences in oral microbiome diversity, immune responses, and dietary habits. Additionally, anatomical and immunological differences in periodontal tissues limit the direct extrapolation of findings to the human population [71]. Future studies should incorporate human cohort studies or alternative preclinical models that more closely resemble human periodontal disease to improve translational relevance. Another limitation of this study is that only male mice were used, which may limit the generalizability of the findings. Sex differences in the metabolism, immune responses, and microbiome composition could lead to varied dietary effects [72,73,74]. Future studies should include both male and female mice to determine whether sex-specific effects contribute to the observed dietary responses.

Additionally, while we measured a specific set of inflammatory markers, including IFN-γ, IL-1β, IL-6, IL-10, IL-17A, and TNF-α, we did not assess other important inflammatory markers such as C-reactive protein (CRP), which may provide future insights into systemic immune responses. Moreover, baseline microbiome profiling was also not conducted prior to dietary intervention, which may have influenced microbial shifts. Future research should incorporate pre-intervention microbiome assessments to better account for individual variability. Moreover, environmental factors such as cage effects, social interactions, and handling stress, although controlled as much as possible, may have introduced minor variations in microbiome composition and host immune responses. The relatively small sample size may have reduced the statistical power to detect subtle effects, and the study duration may not have been sufficient to observe the long-term impacts of dietary fibre on health outcomes. Additionally, the high-fibre content (40%) may have been excessive under experimental conditions, potentially leading to unintended negative effects on growth and development. Future research should focus on optimising fibre intake levels to balance metabolic benefits while ensuring adequate growth. Larger sample sizes and longer study durations will enhance the robustness of findings. Furthermore, mechanistic studies exploring the interactions between diet, microbiota, immune responses, and systemic metabolism are needed.

Translational research in human populations will be critical to validate these findings and assess the practical implications of dietary fibre intake for metabolic and inflammatory disorders. To enhance translational relevance, future studies should incorporate human cohort studies or alternative preclinical models that better replicate human periodontal disease, accounting for differences in oral microbiome diversity, immune responses, and dietary patterns. Additionally, integrating multi-omics approaches in human studies will be essential to confirm the mechanisms identified in murine models and determine whether similar microbiome and metabolic shifts occur in human populations.

## 5. Conclusions

Our findings demonstrate that a high-fibre diet significantly reduces body weight and fat mass, improves glycaemic control, and alters systemic inflammatory markers, suggesting potential benefits for metabolic and immune regulation. Despite the systemic benefits of high-fibre intake, no significant differences in alveolar bone loss and root length were observed, suggesting that the direct impact of dietary fibre on oral bone health remains unclear. The observed microbial shifts may be influenced by weight loss-induced physiological stress, which warrants further investigation into how dietary fibre modulates host–microbe interactions under different physiological states. These findings reinforce the critical role of dietary fibre in shaping metabolic and inflammatory pathways, while highlighting the need for a deeper understanding of its microbiome-mediated effects. Future research should aim to elucidate the mechanistic pathways linking dietary fibre, inflammation, and host–microbiome interactions, as well as determine optimal fibre intake levels that maximise systemic health benefits while minimising potential unintended consequences.

## Figures and Tables

**Table 1:** Biological parameters and statistical comparison between high- and low-fibre groups at the endpoint.

Biological Parameters Assessed	High-Fibre Group (*n* = 14) (Mean ± Standard Deviation)	Low-Fibre Group (*n* = 17) (Mean ± Standard Deviation)	Statistical Significance (Unpaired *t*-Test)
Body weight (g)	22.9 ± 1.82	27.3 ± 1.53	****
Fat mass (g)	1.53 ± 0.32	2.22 ± 0.63	***
Lean mass (g)	19.60 ± 1.46	23.83 ± 1.14	****
Blood glucose (mg/dL)	6.83 ± 1.56	8.92 ± 1.70	**
Alveolar bone loss (mm)	0.214 ± 0.018	0.211 ± 0.025	ns
Root length (mm)	0.919 ± 0.047	0.925 ± 0.042	ns

** *p* ≤ 0.01, *** *p* ≤ 0.001, **** *p* ≤ 0.0001, and ns = not significant.

## Data Availability

The 16S rRNA gene sequencing files from faecal and oral samples are available in the NCBI Sequence Read Archive (SRA) under BioProject PRJNA1227709 (SUB15108928 and SUB15109197).

## Supplementary Materials

The following supporting information can be downloaded at: https://www.mdpi.com/article/10.3390/nu17071146/s1, Table S1: Compositions of High Fibre (HF SF11-029) and Low Fibre (LF SF13-055) as outlined by Specialty Feeds Western Australia; Table S2: Faecal polar-metabolites analysis data.

## Abbreviations

The following abbreviations are used in this manuscript:

ABL	Alveolar Bone Crest
ANOVA	Analysis of Variance
AUC	Area Under the Curve
BHI	Brain Heart Infusion
BCFA	Branched-Chain Fatty Acid
CEJ	Cementoenamel Junction
CMC	Carboxyl Methyl Cellulose
CFU	Colony-Forming Unit
DADA2	Divisive Amplicon Denoising Algorithm 2
DESeq2	Differential Expression Sequencing 2
DNA	Deoxyribonucleic Acid
EDC	1-Ethyl-3-(3-dimethylaminopropyl) carbodiimide
ELISA	Enzyme-Linked Immunosorbent Assay
FDR	False Discovery Rate
GPCR	G-Protein-Coupled Receptor
HDAC	Histone Deacetylase
HPLC	High-Performance Liquid Chromatography
IL	Interleukin
LC-MS	Liquid Chromatography–Mass Spectrometry
LC-QQQMS	Liquid Chromatography–Triple Quadrupole Mass Spectrometry
MMP	Matrix Metalloproteinase
NPH	3-Nitrophenylhydrazine Hydrochloride
OD	Optical Density
PBS	Phosphate-Buffered Saline
PCoA	Principal Coordinates Analysis
PERMANOVA	Permutational Multivariate Analysis of Variance
QC	Quality Control
QIIME2	Quantitative Insights into Microbial Ecology 2
rRNA	Ribosomal Ribonucleic Acid
SCFA	Short-Chain Fatty Acid
TNF-α	Tumor Necrosis Factor-alpha
