# Supplementary material for: Dietary Fibre Modulates Body Composition, Blood Glucose, Inflammation, Microbiome, and Metabolome in a Murine Model of Periodontitis
Source: Nutrients. 2025 Mar 26;17(7):1146. doi: 10.3390/nu17071146 (PMC11990244; doi:10.3390/nu17071146)
Supplement: Supplementary file 1 [file nutrients-17-01146-s001.zip › nutrients-3522170-supplementary.pdf]

nutrients

MDPI

**Table S1.** Compositions of High Fibre (HF SF11-029) and Low Fibre (LF SF13-055) as outlined by Specialty Feeds Western Australia.

| | | HF (SF11-029) | LF (SF13-055) |
|---|---|---|---|
| **Nutritional Parameters (%)** | Protein | 13.2 | 19.4 |
| | Total Fat | 4.5 | 7 |
| | Crude Fibre | 35 | 2 |
| | AD Fibre | 35 | 2 |
| **Digestible Energy (MJ/Kg)** | | 11 | 17 |
| **Total Digestible Energy (%)** | Lipids | 15 | 15.1 |
| | Protein | 19.7 | 20.1 |
| | Carbohydrates | 65.3 | |
| **Ingredients (g/Kg)** | Casein (Acid) | 126 | 200 |
| | Wheat Starch | 402 | 433 |
| | Canola Oil | 44 | 70 |
| | Cellulose | 200 | 11.5 |
| | Guar Gum | 200 | 11.5 |
| | L Methionine | 1.9 | 3 |
| | Calcium Carbonate | 8.3 | 13.1 |
| | Sodium Chloride | 1.6 | 2.6 |
| | AIN93 Trace Minerals | 0.9 | 1.4 |
| | Potassium Citrate | 1.6 | 2.5 |
| | Potassium Dihydrogen Phosphate | 44 | 6.9 |
| | Potassium Sulphate | 1 | 1.6 |
| | Choline Chloride (75%) | 1.6 | 2.5 |
| | AIN93 Vitamins | 6.3 | 10 |
| | Sucrose | | 100 |
| | Dextrinised Starch | | 132 |

**Table S2.** Faecal polar-metabolites analysis data.

| Metabolite | *p*_Value | Adjusted_*p*_Value | Significance |
|---|---|---|---|
| Adenosine monophosphate_HMDB0000045 | 0.005074868 | 0.040961435 | * |
| Uridine 5'-monophosphate_HMDB0000288 | 0.005074868 | 0.040961435 | * |
| Deoxyinosine_HMDB0000071 | 0.005074868 | 0.040961435 | * |
| Cytidine_HMDB0000089 | 0.005074868 | 0.040961435 | * |
| Ornithine_HMDB0000214 | 0.005074868 | 0.040961435 | * |
| Propionic acid_HMDB0000237 | 0.005074868 | 0.040961435 | * |
| Pyroglutamic acid_HMDB0000267 | 0.005074868 | 0.040961435 | * |
| Xanthine_HMDB0000292 | 0.005074868 | 0.040961435 | * |
| Azelaic acid_HMDB0000784 | 0.005074868 | 0.040961435 | * |
| Suberic acid_HMDB0000893 | 0.005074868 | 0.040961435 | * |
| Citrulline_HMDB0000904 | 0.005074868 | 0.040961435 | * |
| Threonic acid_HMDB0000943 | 0.005074868 | 0.040961435 | * |
| Galactonolactone_HMDB0002541 | 0.005074868 | 0.040961435 | * |
| D-Glucurono-6,3-lactone_HMDB0006355 | 0.005074868 | 0.040961435 | * |
| Inosine_HMDB0000195 | 0.008239019 | 0.04900048 | * |
| Gluconic acid_HMDB0000625 | 0.008239019 | 0.04900048 | * |
| Phosphate_HMDB0001429 | 0.008239019 | 0.04900048 | * |

| | | | |
|---|---|---|---|
| alpha-Tocopherol_HMDB0001893 | 0.008239019 | 0.04900048 | * |
| Erythritol_HMDB0002994 | 0.008239019 | 0.04900048 | * |
| Urocanic acid_HMDB0000301 | 0.013065227 | 0.070303363 | ns |
| 7alpha-Hydroxy-3-oxo-5beta-cholan-24-oic acid_HMDB0000503 | 0.013065227 | 0.070303363 | ns |
| Citric acid_HMDB0000094 | 0.020240571 | 0.095299353 | ns |
| Glutamic acid_HMDB0000148 | 0.020240571 | 0.095299353 | ns |
| Succinic acid_HMDB0000254 | 0.020240571 | 0.095299353 | ns |
| Adenine_HMDB0000034 | 0.030638988 | 0.115406855 | ns |
| 2-Hydroxy-3-methylbutyric acid_HMDB0000407 | 0.030638988 | 0.115406855 | ns |
| Ethylmalonic acid_HMDB0000622 | 0.030638988 | 0.115406855 | ns |
| FAD_HMDB0001248 | 0.030638988 | 0.115406855 | ns |
| Itaconic acid_HMDB0002092 | 0.030638988 | 0.115406855 | ns |
| Galacturonic acid_HMDB0002545 | 0.030638988 | 0.115406855 | ns |
| Alanine_HMDB0000161 | 0.045327562 | 0.142278181 | ns |
| Dodecanoic acid_HMDB0000638 | 0.045327562 | 0.142278181 | ns |
| Pentadecanoic acid_HMDB0000826 | 0.045327562 | 0.142278181 | ns |
| Rhamnose_HMDB0000849 | 0.045327562 | 0.142278181 | ns |
| NAD_HMDB0000902 | 0.045327562 | 0.142278181 | ns |
| 3-Aminoisobutanoic acid_HMDB0003911 | 0.045327562 | 0.142278181 | ns |
| Valine_HMDB0000883 | 0.065552161 | 0.180668152 | ns |
| alpha-Ketoisovaleric acid_HMDB0000019 | 0.065552161 | 0.180668152 | ns |
| Malonic acid_HMDB0000691 | 0.065552161 | 0.180668152 | ns |
| Erucic acid_HMDB0002068 | 0.065552161 | 0.180668152 | ns |
| Cysteic acid_HMDB0002757 | 0.065552161 | 0.180668152 | ns |
| Proline_HMDB0000162 | 0.092695803 | 0.23276946 | ns |
| Pantothenic acid_HMDB0000210 | 0.092695803 | 0.23276946 | ns |
| Acetylglycine_HMDB0000532 | 0.092695803 | 0.23276946 | ns |
| D-Lactic acid_HMDB0001311 | 0.092695803 | 0.23276946 | ns |
| Sorbitol_HMDB0000247 | 0.128205275 | 0.268281409 | ns |
| 2-Ketobutyric acid_HMDB0000005 | 0.128205275 | 0.268281409 | ns |
| Glyceric acid_HMDB0000139 | 0.128205275 | 0.268281409 | ns |
| Oxoglutaric acid_HMDB0000208 | 0.128205275 | 0.268281409 | ns |
| Sepiapterin_HMDB0000238 | 0.128205275 | 0.268281409 | ns |
| Hexadecanedioic acid_HMDB0000672 | 0.128205275 | 0.268281409 | ns |
| Ketoleucine_HMDB0000695 | 0.128205275 | 0.268281409 | ns |
| N-Acetyltyrosine_HMDB0000866 | 0.128205275 | 0.268281409 | ns |
| 12-Hydroxydodecanoic acid_HMDB0002059 | 0.128205275 | 0.268281409 | ns |
| Indoleacetic acid_HMDB0000197 | 0.173485468 | 0.326730965 | ns |
| 3-Methyladipic acid_HMDB0000555 | 0.173485468 | 0.326730965 | ns |
| Leucine_HMDB0000687 | 0.173485468 | 0.326730965 | ns |
| Methionine_HMDB0000696 | 0.173485468 | 0.326730965 | ns |
| N-Acetylalanine_HMDB0000766 | 0.173485468 | 0.326730965 | ns |
| Myristic acid_HMDB0000806 | 0.173485468 | 0.326730965 | ns |
| Serine_HMDB0000187 | 0.22976627 | 0.405681071 | ns |
| Orotic acid_HMDB0000226 | 0.22976627 | 0.405681071 | ns |
| N-alpha-Acetyllysine_HMDB0000446 | 0.22976627 | 0.405681071 | ns |
| Creatinine_HMDB0000562 | 0.22976627 | 0.405681071 | ns |
| Biotin_HMDB0000030 | 0.297953062 | 0.480981371 | ns |

| | | | |
|---|---|---|---|
| Glycine_HMDB0000123 | 0.297953062 | 0.480981371 | ns |
| Fructose_HMDB0000660 | 0.297953062 | 0.480981371 | ns |
| Undecanoic acid_HMDB0000947 | 0.297953062 | 0.480981371 | ns |
| Maltitol_HMDB0002928 | 0.297953062 | 0.480981371 | ns |
| N-Acetylmethionine_HMDB0011745 | 0.297953062 | 0.480981371 | ns |
| Glucose_HMDB0000122l | 0.378477593 | 0.562736422 | ns |
| 3-Hydroxybutyric acid_HMDB0000011 | 0.378477593 | 0.562736422 | ns |
| para-Hydroxyphenylacetic acid_HMDB0000020 | 0.378477593 | 0.562736422 | ns |
| Butyric acid_HMDB0000039 | 0.378477593 | 0.562736422 | ns |
| N-Acetyl-D-glucosamine_HMDB0000215 | 0.378477593 | 0.562736422 | ns |
| 3-Methylglutaconic acid_HMDB0000522 | 0.378477593 | 0.562736422 | ns |
| Glycerol_HMDB0000131 | 0.471169998 | 0.619095463 | ns |
| Malic acid_HMDB0000156 | 0.471169998 | 0.619095463 | ns |
| Phenylalanine_HMDB0000159 | 0.471169998 | 0.619095463 | ns |
| Aspartic acid_HMDB0000191 | 0.471169998 | 0.619095463 | ns |
| Thymine_HMDB0000262 | 0.471169998 | 0.619095463 | ns |
| Sulfolithocholic acid_HMDB0000907 | 0.471169998 | 0.619095463 | ns |
| Tryptophan_HMDB0000929 | 0.471169998 | 0.619095463 | ns |
| S-Adenosylhomocysteine_HMDB0000939 | 0.471169998 | 0.619095463 | ns |
| N-Acetylglutamic acid_HMDB0001138 | 0.471169998 | 0.619095463 | ns |
| 2-Hydroxyethanesulfonate_HMDB0003903 | 0.471169998 | 0.619095463 | ns |
| Fumaric acid_HMDB0000134 | 0.575173532 | 0.691432012 | ns |
| Maltose_HMDB0000163 | 0.575173532 | 0.691432012 | ns |
| Lysine_HMDB0000182 | 0.575173532 | 0.691432012 | ns |
| N-Acetylneuraminic acid_HMDB0000230 | 0.575173532 | 0.691432012 | ns |
| Pyruvic acid_HMDB0000243 | 0.575173532 | 0.691432012 | ns |
| Taurine_HMDB0000251 | 0.575173532 | 0.691432012 | ns |
| Uridine_HMDB0000296 | 0.575173532 | 0.691432012 | ns |
| alpha-Linolenic acid_HMDB0001388 | 0.575173532 | 0.691432012 | ns |
| Threonine_HMDB0000167 | 0.688920556 | 0.770772503 | ns |
| Palmitic acid_HMDB0000220 | 0.688920556 | 0.770772503 | ns |
| Ribose_HMDB0000283 | 0.688920556 | 0.770772503 | ns |
| Pelargonic acid_HMDB0000847 | 0.688920556 | 0.770772503 | ns |
| 3-Methylxanthine_HMDB0001886 | 0.688920556 | 0.770772503 | ns |
| Oxalic acid_HMDB0002329 | 0.688920556 | 0.770772503 | ns |
| Raffinose_HMDB0003213 | 0.688920556 | 0.770772503 | ns |
| Glycolic acid_HMDB0000115 | 0.810181236 | 0.839912658 | ns |
| 4-Hydroxybenzoic acid_HMDB0000500 | 0.810181236 | 0.839912658 | ns |
| Glutamine_HMDB0000641 | 0.810181236 | 0.839912658 | ns |
| Octadecanedioic acid_HMDB0000782 | 0.810181236 | 0.839912658 | ns |
| 7-Methylguanine_HMDB0000897 | 0.810181236 | 0.839912658 | ns |
| Behenic acid_HMDB0000944 | 0.810181236 | 0.839912658 | ns |
| Nicotinic acid_HMDB0001488 | 0.810181236 | 0.839912658 | ns |
| Arachidic acid_HMDB0002212 | 0.810181236 | 0.839912658 | ns |
| Kynurenic acid_HMDB0000715 | 0.936186293 | 0.9445451 | ns |
| Ethylmethylacetic acid_HMDB0002176 | 0.936186293 | 0.9445451 | ns |
| N-Acetylglutamine_HMDB0006029 | 0.936186293 | 0.9445451 | ns |
| Hypoxanthine_HMDB0000157 | 1 | 1 | ns |
